# Hydroxycholesterol Levels in the Serum and Cerebrospinal Fluid of Patients with Neuromyelitis Optica Revealed by LC-Ag^+^CIS/MS/MS and LC-ESI/MS/MS with Picolinic Derivatization: Increased Levels and Association with Disability during Acute Attack

**DOI:** 10.1371/journal.pone.0167819

**Published:** 2016-12-12

**Authors:** Eunju Cha, Kang Mi Lee, Ki Duk Park, Kyung Seok Park, Kwang-Woo Lee, Sung-Min Kim, Jaeick Lee

**Affiliations:** 1 Doping Control Center, Korea Institute of Science and Technology, Seoul, Korea; 2 Brain Science Institute, Korea Institute of Science and Technology, Seoul, Korea; 3 Department of Neurology, Seoul National University Bundang Hospital, Gyeonggi-do, Korea; 4 Department of Neurology, College of Medicine, Seoul National University, Seoul, Korea; Heinrich-Heine-Universitat Dusseldorf, GERMANY

## Abstract

Neuromyelitis optica (NMO) is an inflammatory demyelinating disease of the central nervous system (CNS). Hydroxycholesterols (OHCs), metabolites of CNS cholesterol, are involved in diverse cellular responses to inflammation and demyelination, and may also be involved in the pathogenesis of NMO. We aimed to develop a sensitive and reliable method for the quantitative analysis of three major OHCs (24S-, 25-, and 27-OHCs), and to evaluate their concentration in the cerebrospinal fluid (CSF) and serum of patients with NMO. The levels of the three OHCs in the serum and CSF were measured using liquid chromatography-silver ion coordination ionspray tandem mass spectrometry and liquid chromatography-electrospray ionization tandem mass spectrometry with picolinyl ester derivatization, respectively. The linear range was 5–250 ng/mL for 24S- and 27-OHC, and 0.5–25 ng/mL for 25-OHC in serum, and was 0.1–5 ng/mL for 24S- and 27-OHC, and 0.03–1 ng/mL for 25-OHC in CSF. Precision and accuracy were 0.5%–14.7% and 92.5%–109.7%, respectively, in serum, and were 0.8%–7.7% and 94.5%–119.2%, respectively, in CSF. Extraction recovery was 82.7%–90.7% in serum and 68.4%–105.0% in CSF. When analyzed in 26 NMO patients and 23 control patients, the 25-OHC (0.54 ± 0.96 ng/mL vs. 0.09 ± 0.04 ng/mL, *p* = 0.032) and 27-OHC (2.68 ± 3.18 ng/mL vs. 0.68 ± 0.25 ng/mL, *p* = 0.005) were increased in the CSF from NMO patients. When we measured the OHC_CSF_ index that controls the effects of blood–brain barrier disruption on the level of OHC in the CSF, the 27-OHC_CSF_ index was associated with disability (0.723; 95% confidence interval (CI)– 0.181, 0.620; *p* = 0.002), while the 24-OHC_CSF_ index (0.518; 95% CI– 1.070, 38.121; *p* = 0.040) and 25-OHC_CSF_ index (0.677; 95% CI– 4.313, 18.532; *p* = 0.004) were associated with the number of white blood cells in the CSF of NMO patients. Our results imply that OHCs in the CNS could play a role in the pathogenesis of NMO.

## Introduction

Neuromyelitis optica (NMO) is considered to be the first inflammatory demyelinating disease of the central nervous system (CNS) caused by an identified autoantibody [1,2]. In the past, NMO was frequently misdiagnosed as multiple sclerosis (MS), mostly due to its relapsing and remitting disease course and demyelination in the CNS [3]. However, the discovery of a disease-specific autoantibody to aquaporin4 (AQP4-Ab) revealed that NMO is different from MS in that it has a more severe clinical course, distinct pathologic findings, and different responses to treatment [2,4,5]. The exact pathomechanism of NMO is still unclear, but disease-specific AQP4-Abs are thought to be responsible for NMO by causing the activation of complement and/or natural killer cells [6], inflammation, demyelination [7], eosinophils recruitment [8], and astrocytic necrosis [9].

Cholesterol is a major component of the CNS, undergoes side chain oxidizations, and is metabolized as some types of OHCs. These OHCs can modulate sex hormone receptors that prevent inflammation and/or demyelination of the brain [10], upregulate chemotactic cytokines that recruit eosinophils and natural killer cells [11,12], mediate glutamate excitotoxicity [13], and induce neuronal necrosis [14]. These mechanisms are major components in the pathogenesis of NMO [2,8].

The rapid and accurate quantitative determination of 24S-, 25- and 27-OHC in biological fluids such as serum and cerebrospinal fluid (CSF) is highly challenging due to their low concentration. For example, in the brain, the ratio of cholesterol to OHCs varies from 500:1 to 1000:1 [15]. Moreover, the concentration of OHCs is approximately 100-fold lower in CSF compared with serum. The simultaneous quantitative analysis of 24S-, 25-, and 27-OHC is challenging not only because of sensitivity but also due to the difficulties in obtaining complete chromatographic separation. Because OHCs have the same molecular weights and similar structures, complete chromatographic separation is necessary. Thus, a highly sensitive and selective analytical method is essential for the simultaneous quantitative determination of the OHC concentration.

Over several years a variety of methods for the quantitative analysis of OHCs in biological fluids have been reported [16–22]. The most established methods are based on gas or liquid chromatography coupled with mass spectrometry. Gas chromatography (GC)-based analytical methods, generally require long analysis times, laborious processes, and derivatization steps. In particular, the derivatization step is time-consuming and does not always produce satisfactory derivatives. Although GC-based methods have some disadvantages, the Gas chromatography-electron impact/tandem mass spectrometry (GC-EI/MS/MS) method yielded excellent sensitivity and selectivity for OHCs [16]. Liquid chromatography (LC)-based analytical methods reduce analysis time and do not require a derivatization step. Despite these advantages, the LC-based methods suffer from low ionization efficiency under electrospray ionization (ESI) conditions and poor chromatographic resolution of compounds with similar chemical structures [23,24].

Nevertheless, analytical methods based on liquid chromatography-tandem mass spectrometry (LC-MS/MS) have been rapidly developed in recent decades due to their availability and convenience [25–27], and they are used as an alternative to GC-based methods. In LC-based methods, the absence of acidic or basic groups in the structures of analytes results in low ionization efficiency under ESI. atmospheric pressure chemical ionization (APCI) methods have also used however, they still don’t have enough sensitivity for oxysterols detections [23,24]. To overcome the poor ionization efficiency, we investigated LC-based methods using various chemical derivatizations. Chemical derivatization makes it possible to improve the ionization efficiency of poorly ionizable or non-ionizable substances. In particular, picolinic acid and Grignard P reagents have been commonly used in LC-MS/MS [25–28]. However, the derivatization technique still has notable disadvantages. Therefore, in the present study, the LC-Ag^+^CIS/MS/MS method was investigated to minimize the difficulties in derivatization and to enhance the ionization efficiency. Based on the LC-MS/MS method with Ag^+^CIS ionization, we performed simultaneous quantification of 24S-, 25- and 27-OHC in serum (OHC_serum_). However, this method was limited for the analysis of OHC in the CSF (OHC_CSF_) because OHC_CSF_ is present in only a trace amount compared with OHC_serum_ [29]. To analyze OHC_CSF_, we developed a highly sensitive and selective analytical method using picolinyl ester derivatization (PE). Consequently, we developed serum- and CSF-customized analytical methods for the simultaneous quantitative determination of three major OHCs using LC-Ag^+^CIS/MS/MS or LC-ESI/MS/MS with PE. In addition, we determined whether OHC concentration is increased in the CSF of NMO patients, and assessed whether OHC_CSF_ concentration is associated with disability during acute NMO attacks.

## Materials and Methods

### 1. Materials

24S-Hydroxycholesterol [cholest-5-ene-3-beta,24S-diol], 25-hydroxycholesterol [cholest-5-ene-3-beta,25-diol], 27-hydroxycholesterol [cholest-5-ene-3beta,27-diol], 24(R/S)-hydroxycholesterol-d6 [26, 26, 26, 27, 27, 27-hexadeuterocholest-5-ene-3 beta,24-diol], 25-hydroxycholesterol-d6 [26, 26, 26, 27, 27, 27-hexadeuterocholest-5-ene-3 beta,25-diol] and 27-hydroxycholesterol-d6 [25, 26, 26, 26, 27, 27-hexadeuterocholest-5-ene-3 beta,27-diol] were obtained from Avanti Polar Lipids (Alabama, USA). 2-methyl-6-nitrobenzoic anhydride, 4-dimethylaminopyridine, picolinic acid, pyridine and trimethylamine were purchased from Sigma Aldrich Co. (St. Louis, USA). Phosphoric acid and potassium hydroxide were provided by Junsei (Tokyo, Japan). Distilled water was purified using a Milli-Q purification system (Millipore, Massachusetts, USA). High performance liquid chromatography (HPLC)-grade methanol, ethanol, hexane and 2-propanol were purchased from Burdick & Jackson (Ulsan, Korea).

### 2. NMO disease

#### 2.1. Patients

This study included 26 NMO patients who had positive AQP4-Ab tests, met the revised diagnostic criteria for NMO or NMO spectrum disorders [30,31], visited the Seoul National University Hospital and Seoul National University Bundang Hospital between January 2010 and April 2013, and gave written consent. The disability of NMO patients was graded using the Kurtzke extended disability status scale (EDSS) during the acute stage (within 30 days of an attack) [32]. Serum from NMO patients was tested for the presence of AQP4-Ab at the Weatherall Institute of Molecular Medicine (John Radcliffe Hospital, Oxford, UK) using a cell-based assay as described previously [33]. Serum and CSF samples were obtained from NMO patients during the acute stage and before the initiation of steroid pulse treatment [34]. The number of white blood cells (WBC) and levels of protein, glucose, albumin, and IgG were also assessed in the CSF of patients.

The control group comprised 23 age- and sex-matched patients who had neurological diseases other than inflammatory or degenerative CNS diseases (14 polyneuropathy, 3 cranial nerve palsy, 1 inflammatory myositis, 1 radiculopathy, 1 spinal arteriovenous malformation, 1 somatoform disorder, 1 plexopathy, and 1 spondylosis), did not receive steroid treatment, and gave written consent. The basal characteristics and CSF findings of included subjects are described in Table 1.

**Table 1:** Basal characteristics and cerebrospinal fluid findings.

	Groups		*p–value*
	NMO	Controls	
**Basal characteristics**			
Number	26	23	
Age at sampling (years)	51.9 ± 7.4 (37.1 − 72.8)	46.9 ± 15.9 (20 − 67.8)	n.s.
Number of males (%)	7 (27%)	8 (35%)	n.s.
Body mass index	22.8 ± 2.7 (19 − 28.9)	23.2 ± 2.7 (17.4 − 28.2)	n.s.
Serum total cholesterol (mg/dL)	187.2 ± 59.9 (65 − 320)	168.8 ± 31.3 (110 − 225)	n.s.
Serum glucose (mg/dL)	121 ± 41 (77 − 158)	94 ± 14.3 (78 − 113)	n.s.
**Cerebrospinal fluid study**			
White blood cell (cells /mm^3^)	10.6 ± 17.7 (0 − 60)	1.9 ± 6.3 (0 − 30)	0.015
Protein (mg/dL)	42.1 ± 15.4 (20 − 80)	35.5 ± 13.6 (21 − 80)	n.s.
Glucose (mg/dL)	64 ± 10.6 (44 − 88)	62.7 ± 9.2 (42 − 81)	n.s.
Albumin (mg/L)	35.1 ± 22.6 (10.8 − 94.2)	25.5 ± 15.8 (6.4 − 63.5)	n.s.
IgG (mg/L)	6.4 ± 5.2 (1.8 − 22)	4.9 ± 3.7 (1.7 − 15.6)	n.s.

#### 2.2. OHC_CSF_ index in NMO

Patients who have severe attacks of NMO can experience disruption of the blood brain barrier (BBB) [34]. Considering that some OHCs, such as 27-OHC, can enter into the CNS from the circulation, the disruption of the BBB in severe NMO patients may affect the CSF OHC concentration. Therefore, we measured the CNS-synthesized OHCs by calculating the OHC_CSF_ index, which can control the effect of the BBB disruption on the level of OHC_CSF_, using a previous formula with minor modifications [35], as follows: OHC_CSF_ index = (CSF OHC / serum OHC)/(CSF albumin / serum albumin).

#### 2.3. Sampling and storage protocol

Serum and CSF were collected on the same day during the acute disease stage (within 30 days of acute attack onset), and before the initiation of steroid pulse or plasmapheresis treatment [34]. Samples were immediately centrifuged after collection and stored at −80°C. The collection and storage of samples followed recent biobank consent protocols [36] and were also in accordance with the sampling/storage protocol from a previous study on the level of OHCs in the CSF and serum of patients [37]. For the quantitation method, we produced quality control samples (QCs) that were analyzed with every batch, repeatedly monitored to evaluate the formation/degradation of OHCs, and assessed as stable.

#### 2.4. Statistical analysis

To compare the groups, the non-parametric Mann–Whitney U–test was used, and the results were expressed as means ± standard deviation. Univariate and multivariate stepwise linear regression analyses were used to determine the association between the values. Adjusted values were used for confidence intervals. The Predictive Analytics Software (PASW) was used for all statistics (ver. 18; SPSS Inc., Chicago, IL, USA); moreover, *p* values < 0.001 were considered to indicate statistical significance.

#### 2.5. Standard protocol approval, registration, and patient consent

This study was approved by the Institutional Review Board of Seoul National University Hospital and Seoul National University Bundang Hospital (IRB number: H-1012-023-317 and B-1007-105-401, respectively). All patients provided an informed written consent prior to participation.

### 3. Sample analysis and instruments

d_6_-25-Hydroxycholesterol was added to 500 μL of serum and CSF as an internal standard, and alkaline hydrolysis was performed in 2 mL of 1 N ethanolic KOH at 50°C for 2 h. The hydrolyzed sample was subsequently neutralized to pH 7 with 75 μL of phosphoric acid. The mixture was then centrifuged for 5 min at 1000 g and the clear supernatant was collected for subsequent solid phase extraction (SPE). A large amount of endogenous substance such as cholesterol, can give rise to poor chromatographic separation and ion suppression. Therefore, SPE was used for the pre-concentration of OHCs and clean-up of interferences such as cholesterol from the matrix. We modified the sample preparation using SPE based on previous research [38]. In our SPE process, cholesterol was monitored using the multiple reaction monitoring (MRM) mode to evaluate the removal of cholesterol from the matrix, and we found that most of the cholesterol was removed. As a result, serious effects on chromatographic separation (interference peaks) and ion suppression by cholesterol were not observed. It was also checked for validation and sample analysis. The sample was loaded onto an Oasis HLB extraction cartridge (150 mg, Waters Corporation, Massachusetts, USA) that had been preconditioned with 1 mL of hexane/2-propanol (50:50, v/v), 1 mL of methanol, and 2 mL of water. The extraction cartridge was washed with 4 mL of methanol/water (75/25, v/v) and briefly dried under vacuum. The analyte was then eluted with 3 mL of hexane/2-propanol (50:50, v/v) using gravity, and was evaporated to dryness with an evaporator (Rotavapor, Buchi, Flawil, Switzerland) at 35°C. For serum, the residue was dissolved in 100 μL of methanol, and 2 μL was injected into the LC-MS/MS system. In addition, several steps were added for the derivatization of CSF OHC (OHC_CSF_). For OHC_CSF_, cholesterol removed CSF was converted to picolinic acid derivatives and was evaluated as blank for calibration. As a result, exogenous 24-, 25- and 27-OHCs were not observed in the blank CSF. For PE, a reagent mixture was prepared with 2-methyl-6-nitrobenzoic anhydride (10 mg), 4-dimethylaminopyridine (3 mg), picolinic acid (8 mg), pyridine (150 μL), and triethylamine (20 μL). The freshly prepared reagent mixture (170 μL) was added to the dried residue and incubated at 80°C for 1 h to derivatize. After the addition of 1 mL of hexane, the mixture was vortexed and centrifuged for 5 min at 1000 *g*. The clear supernatant was collected and evaporated at 80°C under a steam of nitrogen gas. The residue was dissolved in 50 μL of methanol, and 2 μL were injected into the LC-MS/MS system.

LC-MS/MS analyses were performed using an API 4000 triple-quadrupole mass spectrometer (AB Sciex, Toronto, Canada) equipped with an electrospray ionization (ESI) source, in the positive ionization mode. The electrospray source was coupled online with a Shimadzu UFLC system (Shimadzu Corporation, Kyoto, Japan). The mass spectrometer operated with a heated nebulizer interface in a positive ionization mode at high mass resolution for both Q1 and Q3. Air was used as the nebulizer gas, and nitrogen was used as the curtain and collision gas. For serum, MRM transitions for the *m/z* 509–491 and *m/z* 515–497 channels were employed for the three OHCs and internal standard (IS), respectively, with a 100 ms dwell time per channel and 5 ms pause between channels. For CSF, the *m/z* 635–512 and *m/z* 641–518 channels were used for the OHCs and IS, respectively.

The optimized acquisition parameters were set at the following: ion spray voltage 5400 V, nebulizer gases (GS1 and GS 2) 60 psi, curtain gas psi, (CUR) 20 psi, collision gas (CAD) 4 units, source temperature (TEM) 650°C, declustering potential (DP) 141 V for serum; 70 V for CSF, entrance potential (EP) 10 V, collision energy (CE) 35 eV for serum; 32 eV, 30 eV and 38 eV for 24S-OHC, 25-OHC and 27-OHC, respectively, for CSF, and collision cell exit potential (CXP) 12 V for serum and 14 V for CSF.

For serum, an ACE C18 column (150 × 2.1 mm, 3 μm) at 30°C was used. The LC mobile phases comprised 50 μM silver acetate in distilled water (solvent A), and 50 μM silver acetate in methanol (solvent B). Gradient elution was performed according to the following elution program: 0–0.1 min: 50–89% B, 0.1–14.5 min: 89–92%B, 14.5–15.0 min: 92–100% B, 15.0–26.0 min: 100% B and 26.1–30 min: 50% B at a flow late of 0.2 mL/min. For CSF, a C18 Kinetex column (100 × 2.10 mm, 2.6 μm) at 35°C was used. The mobile phases, consisted of 0.01% formic acid in distilled water (solvent A), and 0.01% formic acid in methanol (solvent B), and were used with a gradient elution of A:B = 50;50 to 10:90 (0–0.1 min), 10:90 to 5:95 (0.1–9.0min), 5:95 to 0:100 (9.0–9.10 min), 0:100 (9.10–13.0 min) and 50:50 (13.1–15.0 min) at a flow late of 0.35 mL/min.

### 4. Calibration sample for validation

For validation, cholesterol stripped serum and CSF were used because each OHC is normally present in human samples at different concentrations. The cholesterol-stripped sample was prepared by solid-phase extraction. The Oasis HLB extraction cartridge was preconditioned with 5 mL of methanol and 4 mL of water, 500 μL of serum or CSF were then loaded, and the eluent without solvent using gravity was used as the cholesterol-stripped sample. Eluents were checked before analysis to ensure that the cholesterol had been completely removed. They were freshly prepared and used for calibration curves after the removal of cholesterol from the serum and CSF. Calibration standards and quality control samples were fortified with standard solutions of 24S-, 25- and 27-OHC in the cholesterol stripped serum or CSF, and underwent the same sample preparation procedure.

### 5. Validation methods

#### 5.1. Linearity and limit of quantification

To assess linearity, calibration curves were generated by plotting the peak area ratio of the analyte to the internal standard, versus the concentrations in the standard-spiked serum samples. Each calibration curve was generated using more than six calibration points and the ranges for each substance are shown in Table 2. The limit of detection (LOD) was evaluated using a signal-to-noise ratio of three, and the limit of quantification (LOQ) was determined as the lowest spiked concentration with a calculated precision of less than 20%.

**Table 2:** Linearity of the calibration curve, LOD and LOQ for OHCs.

Analytes	Range (ng/mL)	LOD (ng/mL)	LOQ (ng/mL)	*R*^*2*^
**Serum**				
24S-OHC	5–250	0.3	5	0.9990
25-OHC	0.5–25	0.03	0.5	0.9972
27-OHC	5–250	0.3	5	0.9997
**CSF**				
24S-OHC	0.1–5	0.02	0.1	0.9997
25-OHC	0.03–1	0.02	0.03	0.9998
27-OHC	0.1–5	0.01	0.1	0.9999

#### 5.2. Precision and accuracy

The concentrations of the low-, medium- and high-quality control samples were processed; five replicates in the same run (intra-day accuracy and precision), and five replicates in five separated runs (inter-day accuracy and precision) were analyzed. Precision was calculated as the coefficient of variation of the assayed concentrations. Accuracy was expressed as the bias of the assayed concentration to the expected value. The lower limit of quantification, low, medium and high QC concentrations for each substance are shown in Table 3.

**Table 3:** Results of accuracy, precision and recovery of method validation using quality control samples.

Analytes	QC (ng/mL)	Intra-day (n = 5)		Inter-day (n = 5)		Recovery (%)
		Accuracy (%)	Precision (%)	Accuracy (%)	Precision (%)	
**Serum**						
24S-OHC	5	96.0	4.7	97.7	10.4	89.8
	10	95.0	4.1	99.7	4.5	88.1
	100	106.0	0.5	100.9	3.0	85.8
	200	99.1	0.9	100.1	1.2	82.7
25-OHC	0.5	109.7	8.2	93.3	14.7	90.7
	1	98.6	5.5	92.5	7.2	89.7
	10	103.0	0.5	105.1	4.1	87.5
	20	94.9	0.9	97.4	2.2	83.7
27-OHC	5	104.5	8.2	107.3	7.5	85.8
	10	103.8	5.5	107.4	4.2	87.5
	100	99.7	0.5	96.0	1.9	88.7
	200	99.7	0.8	101.1	1.0	84.0
**CSF**						
24S-OHC	0.1	97.1	7.6	96.2	4.5	83.7
	0.5	101.6	5.3	108.1	2.3	95.8
	2	98.7	1.4	98.0	1.8	97.2
	5	99.7	1.6	98.5	0.8	92.6
25-OHC	0.03	112.9	7.3	119.2	4.5	68.4
	0.1	107.8	4.0	109.2	3.9	98.5
	0.5	99.6	1.3	101.0	2.3	99.6
	1	107.5	1.2	110.3	3.2	93.2
27-OHC	0.1	115.8	7.7	108.3	3.5	88.2
	0.5	101.3	1.8	100.8	1.6	105.0
	2	97.1	2.3	94.5	2.4	103.2
	5	99.8	1.2	96.3	2.6	97.3

#### 5.3. Extraction recovery

The response of the analyte obtained after the extraction of the quality control samples was compared with the response obtained from the extracted and stripped serum and the CSF matrix enriched with the same amount of analyte after the preparation step. The QC samples at the lower limit of quantification, low, medium, and high concentrations were processed and measured in five replicates. Recovery was determined by comparing the chromatographic peak areas of the serum or CSF samples spiked before extraction with the chromatographic peak areas of serum or CSF samples spiked after extraction.

#### 5.4. Matrix effect

To assess the interference of the serum and the CSF matrix, pooled serum and CSF samples from a large number of people were repeatedly analyzed. To evaluate the matrix effect, an ion suppression test was also performed using a postcolumn infusion method. A stripped sample that had undergone preparation procedure was injected into the column, and the effect of matrix suppression on the response of the infused oxysterols was monitored. As a result, targeted analytes were quantified simultaneously to ensure that there was no interference and ion suppression.

## Results and Discussion

This study simultaneously quantified 24S-, 25-, and 27-OHC in the serum and CSF of patients with NMO using LC-MS/MS. This is the first study to successfully investigate the relationship between three OHCs and NMO disease. A few researches performed the quantification of three OHCs. Nevertheless simultaneous quantitation of three OHCs in serum and CSF is difficult, and it has bottlenecks for sensitive and selective analysis. First, the extremely low concentrations of these OHCs can affect the sensitivity of the analysis. Specifically, the concentration of 25-OHC in CSF is approximately 100-fold lower than that of 24S- and 27-OHC in serum [29]. Second, although chromatography should completely separate the three OHCs, their structural and optical isomeric properties can disturb their separation [39]. Third, endogenous substances in the serum and CSF can lead to selectivity problems because of their similar molecular weights and structures [40]. Therefore, a novel analytical method with high sensitivity and selectivity was essential for the simultaneous quantitation of OHCs.

In our preliminary studies, the measurement of OHC concentration by GC-MS using trimethylsilyl derivatization was attempted. However, the intensity of the TMS derivatized OHCs and the interference of unwanted by-products in the GC-MS system was unsatisfactory. Therefore, an initial effort was made to establish a sensitive and selective method using LC-ESI/MS/MS. Although the conventional ESI is generally used, it is not the best ionization method for OHCs because of its poor ionization efficiency. To overcome this ionization problem, a new method for the enhancement of ionization efficiency using silver ion coordination ionspray was developed. The use of silver ion coordination for the analysis of OHC in serum provided an excellent chromatographic peak shape and sufficient intensity for quantitative analysis. The silver ion is a soft Lewis acid, and carbon–carbon double bonds in unsaturated compounds are soft Lewis bases and serve as likely sites for π complex formation which is charged complex [41]. Bayer et al.[41] showed that cholesterols can be detected as a π complex with Ag^+^ using silver ion coordination ionspray. It was therefore expected that the complex of silver ion-unsaturated OHCs would be detected with mass spectrometry coupled with electrospray ionization (ESI/MS). Van Beek et al. [42] suggested a dual interaction between hydroxyl oxygen and the double bonds, with silver ions. Under the ESI positive mode, the 24S-, 25-and 27-OHC produced the Ag^+^ coordinated molecular ions [M+Ag]^+^ at *m/z* 507.

Despite the high sensitivity attained from the Ag^+^CIS conditions, this method was limited to analysis of the OHCs in serum, because the concentration of OHCs in the CSF is approximately 10-fold lower. To combat this, a highly sensitive analytical method for the analysis of OHCs in CSF was developed using LC-ESI/MS/MS with PE [27,28]. Picolinyl acid can derivatize the hydroxyl group of OHCs, generates [M+2 picolinic acid+Na]^+^ as the abundant ion in ESI positive mode, and is roughly 10-fold more sensitive than Ag^+^CIS. Unfortunately, the PE derivatization method could not be used to determine serum OHC concentrations because, as determined by preliminary studies, there was interference by unknown endogenous interference peaks in most serum samples. However, over the normal range of OHC serum concentrations, the Ag^+^CIS method showed sufficient intensity and a good peak shape, which allowed for the quantification of trace amounts of OHCs. Therefore, highly sensitive and selective analytical methods were developed using silver coordination and PE derivatization for the determination of OHC concentrations in human serum and CSF.

Several studies have been published regarding major OHCs such as 24S-OHC and 27-OHC, but little research on 25-OHC exists due to its low concentration in human biological fluids. Using the Ag^+^CIS method, the signal of 25-OHC was approximately 10-fold higher than that of 24S- and 27-OHC at the same concentration. Therefore, by using this method, the quantification of 25-OHC in human serum is now possible with sufficient sensitivity and selectivity, despite its very low concentration. In addition, PE derivatization showed excellent sensitivity and selectivity with very low LOD (Table 2).

Recently, a highly sensitive GC-MS/MS method for OHC_serum_ has been established [43]. In addition other researches on the quantification of serum and/or CSF has been published. In comparison to the published GC-MS [16–18,21,22], LC-APCI-MS [23, 24] and LC-ESI-MS [27, 28] methods, to the best of our knowledge, the present methods using Ag^+^CIS-MS and PE derivatization provided lower a LOD level, allowing for the quantification of low concentrations of OHCs in serum and CSF. The present Ag^+^CIS and PE analytical methods seem to be the best candidates for the simultaneous quantitation of 24S-, 25-, and 27-OHC in serum and CSF.

### 3.1. LC-MS/MS characteristics of hydroxycholesterols

The stereochemistry of 24-hydroxycholesterol in humans has previously been investigated with emphasis on 24S-hydroxylase expression [44]. In an early report, 24S-hydroxycholesterol was shown to be a single epimer in human biological tissues, and thus the predominant isomer, 24S-hydroxycholesterol, was adopted for the analysis of OHCs.

**Fig 1:**
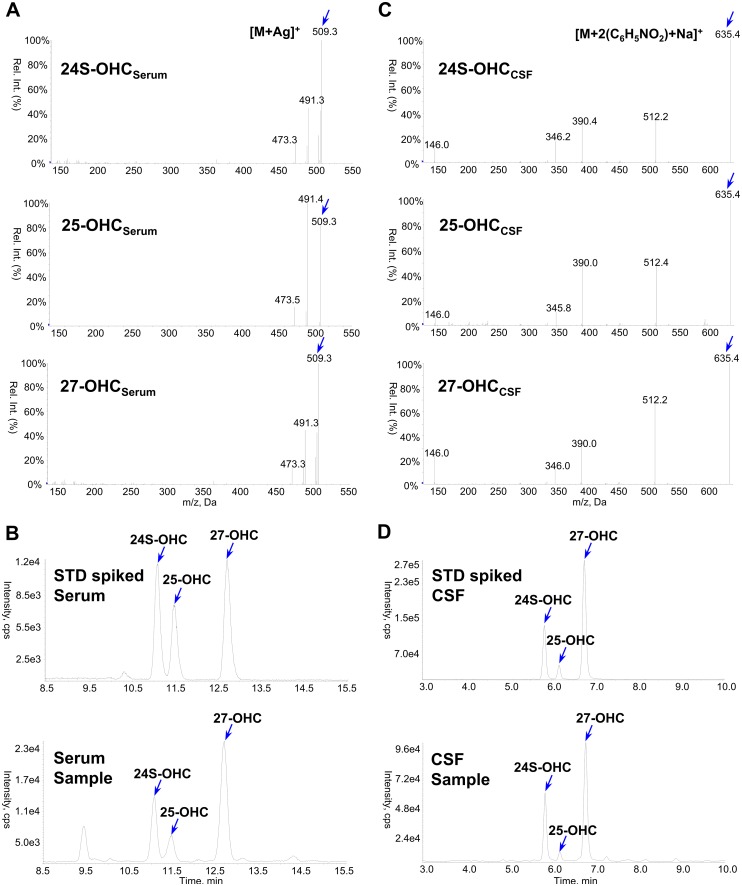
Obtained product ion spectra and representative chromatograms of 24S-, 25- and 27-OHC. For OHC_serum_, spectra (A) and chromatograms (B) by silver coordination. For OHC_CSF_, spectra (C) and chromatograms (D) by picolinyl ester derivatization.

For serum samples, the chromatograms and typical CIS-MS spectra of the three OHCs are shown in Fig 1A and 1B. In the full scan mass spectrum, the observed parent ions are the [M+Ag]^+^ adducts at *m/z* 509 and *m/z* 511, formed by two silver isotopes ^107^Ag and ^109^Ag that are present in a ~ 1:1 ratio [45]. For the IS, [M+Ag]^+^ adducts at *m/z* 515 and *m/z* 517 were observed. As the collision energy voltage was increased, [M+^107^Ag]^+^ at *m/z* 509 gave rise to a fragment ion at *m/z* 491 [M+Ag-H_2_O]^+^ (Fig 1A), and [M+^109^Ag]^+^ at *m/z* 511 gave rise predominantly to a corresponding fragment ion at m/z 493 [M+Ag-H_2_O]^+^. The dominant product of the IS complex was the loss of the water molecule, *m/z* 515 gave a fragment ion at *m/z* 497, and *m/z* 517 gave a fragment ion at *m/z* 499. The Q3 product ion at *m/z* 491 of the target analytes was selected for quantitative SRM analysis after considering interference, signal-to-noise ratio, and sensitivity. As shown in the representative chromatograms of the standard spiked sample and human serum (Fig 1B) obtained from this method, 24S-, 25- and 27-OHC were observed with the same retention.

For the CSF samples, the three OHCs were converted into corresponding picolinyl ester derivatives, which were successfully analyzed by LC-ESI/MS/MS, consequently, highly sensitive and selective results were obtained. The PE derivatives generated [M+2 picolinic acid +Na]^+^ ions as the base peaks under ESI positive conditions. The fragmentation pattern was examined under various levels of collision energy, as a result, a [M+picolinic acid+Na]^+^ (*m/z* 512) ion was observed as the predominant ion, but a [picolinic acid+Na]^+^ (*m/z* 146) ion was also present. Therefore, the [M+2 picolinic acid+Na]^+^ (*m/z* 635) and [M+picolinic acid+Na]^+^ (*m/z* 512) ions were selected as a monitoring ion pair (Q1/Q3) for OHC derivatives (Fig 1C). Fig 1D shows the typical MRM chromatograms obtained by monitoring their transitions to picolinyl derivatives. In the methods developed in this study, MRM analysis allowed for accurate sample quantification with lower limits of quantitation (LLOQ) of 5 ng/mL for 24S- and 27-OHC, and 0.5 ng/mL for 25-OHC in serum, and 0.1 ng/mL for 24S- and 27-OHC, and 0.03 ng/mL for 25-OHC in CSF.

### 3.2. Validation

The validation results for the quantification of three OHCs are summarized in Tables 2 and 3. For serum, the calibration curves of 24S-OHC and 27-OHC were evaluated in the range of 5–250 ng/mL, and for 25-OHC, the range was from 0.5 to 25 ng/mL. Each calibration curve ranged over the various concentrations of OHCs in human serum. For CSF, the calibration curves of 24S-OHC and 27-OHC were evaluated in the range of 0.1–5 ng/mL and for 25-OHC, the range was from 0.03 to 1 ng/mL due to the trace amounts in the CSF. The peak area ratio of each analyte and deuterated internal standard was fitted to a weightless least-squares model to produce the calibration curve, and the linearity was determined by a correlation coefficient (*R*^2^). The linearity of all the calibration curves was higher than 0.99. Intra- and inter-day precision for the target analytes were between 0.5% and 14.7%, and the accuracy ranged from 92.5% to 119.2%. Table 2 shows the LOD and the LOQ for each OHC. The greatest sensitivity was found for 25-OHC. The extraction recoveries ranged between 82.7% and 105.0% (except for 0.03 ng/mL of OHC_CSF_), which are appropriate for such a technique. Coefficients of variation for values obtained at the four concentrations were lower than 10% (data not shown), which meant that the OHC concentration over the range analyzed did not affect recovery in CSF or serum. Mean recovery data are shown in Table 3.

Matrix effects can significantly affect the ionization of the analyte by causing a reduction of the MS/MS response. Therefore, the ion chromatograms of the pooled serum and CSF were repeatedly examined to investigate potential interference. No interference and ion suppression were observed in the human serum and CSF samples at the retention times of the OHCs. In addition, no carryover effect was observed during multiple injections of serum and CSF samples when the instrument was run in batch mode.

### 3.3. Increased OHC in the CSF of NMO patients

**Fig 2:**
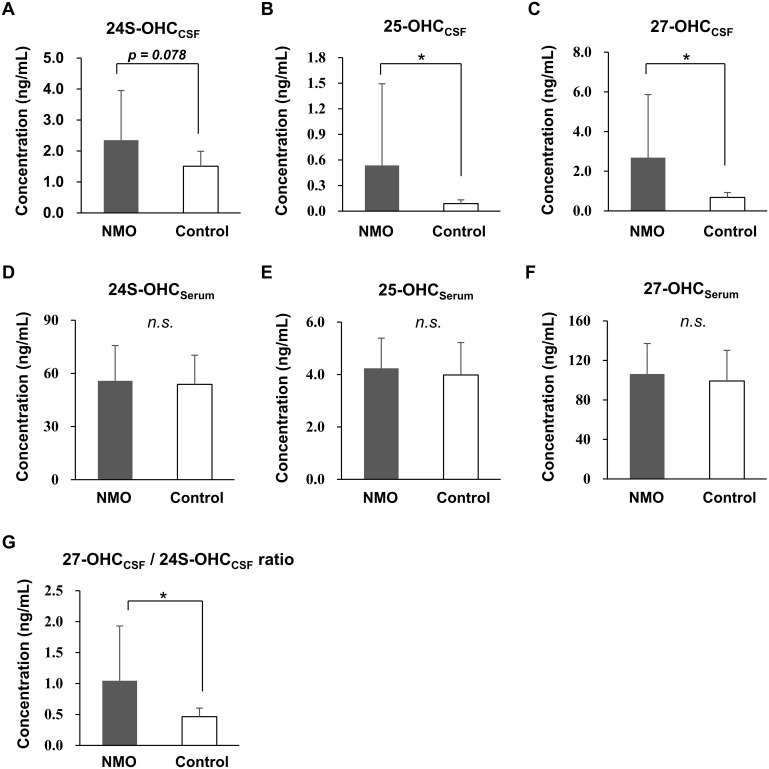
Levels of 24S-, 25-, and 27-OHC in the CSF and serum of patients. Among the levels of OHC_CSF_ (A-C) and OHC_serum_ (D-F), the levels 25- and 27-OHC_CSF_ were increased in patients with NMO compared with controls (B and C). The levels of 24S-OHC_CSF_ (A) and OHC_Serum_ levels did not differ between groups (D–F). The ratio of 27-OHC_CSF_ over 24S-OHC_CSF_, which could be associated with either the disruption of the BBB or increased synthesis of 27-OHC in the CNS, was also increased in the NMO group (G). *p < 0.001; *n*.*s*. = not significant.

Patients with NMO had significantly higher levels of 25-OHC_CSF_ (NMO, 0.536 ng/mL ± 0.957 vs. control, 0.088 ng/mL ± 0.044, *p* < 0.001) and 27-OHC_CSF_ (NMO, 2.684 ng/mL ± 3.180 vs. control, 0.679 ng/mL ± 0.247, *p* < 0.001), compared with controls. However, levels of 24S-OHC_CSF_ (NMO, 2.349 ng/mL ± 1.600 vs. control, 1.509 ng/mL ± 0.481, *p* = 0.078), 24S-OHC_Serum_ (NMO, 55.823 ng/mL ± 19.883 vs. control, 53.809 ng/ml ± 16.443, *p* = 0.703), 25-OHC_Serum_ (NMO, 4.238 ng/mL ± 1.150 vs. control, 3.983 ng/mL ± 1.238, *p* = 0.457), and 27-OHC_Serum_ (NMO, 106.277 ng/mL ± 30.817 vs. control, 99.152 ng/ml ± 31.001, *p* = 0.425) did not differ significantly between the groups. The ratio of 27-OHC to 24S-OHC (27-OHC_CSF_/24S-OHC_CSF_ ratio) in the CSF, which could represent either the degree of BBB disruption [46] or increased 27-OHC synthesis in the CNS, was also moderately increased in patients with NMO compared to controls (NMO, 1.046 ± 0.885 vs. control, 0.464 ± 0.139, *p* < 0.001) (Fig 2).

### 3.4. Association of OHC_CSF_ with disease disability and the number of inflammatory cells in the CNS

**Fig 3:**
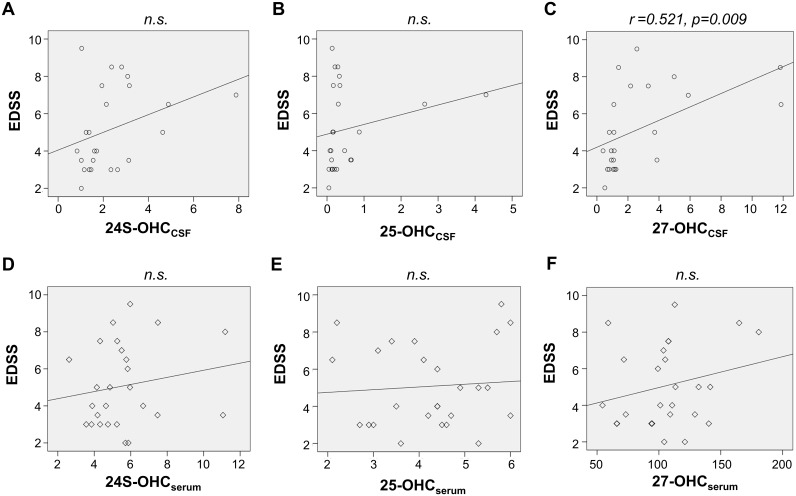
Association of the level of OHCs with disability at acute attack in NMO patients. Of the levels of OHC_CSF_ (A–C) or OHC_Serum_ (D–F) of patients with NMO, only the levels of 27-OHC_CSF_ were significantly associated with their disability at acute attacks (C). *n*.*s*. = not significant.

We assessed the association of OHC_CSF_ of OHC_serum_ with the disability of NMO patients at their acute attack. Univariate linear regression analysis revealed that, among these OHCs, only the level of 27-OHC_CSF_ was significantly associated with disability during acute attack (EDSS) (0.521; 95% CI– 0.100, 0.626; *p* = 0.009) (Fig 3).

To control any confounding effect due to potential BBB disruption and subsequent diffusion of 27-OHC from the serum into the CSF, multivariate analysis for the CSF/serum quotient of albumin (*Q*alb) was conducted. Multivariate regression analysis revealed that only 27-OHC_CSF_, but not *Q*alb, which represents BBB disruption, was significantly associated with the EDSS of NMO patients (Table 4).

**Table 4:** Multivariable analysis for the association with EDSS.

Variables	*β* (95% CI^b^)	t	*p–value*
**27-OHC_CSF_**	0.935 (0.175–1.378)	2.713	0.014
***Q***_**alb**_^a^	–0.589 (–0.489–0.050)	–1.710	0.105

^a^CSF/serum quotient of albumin (*Q*_alb_).

^b^Confidence interval (CI).

**Fig 4:**
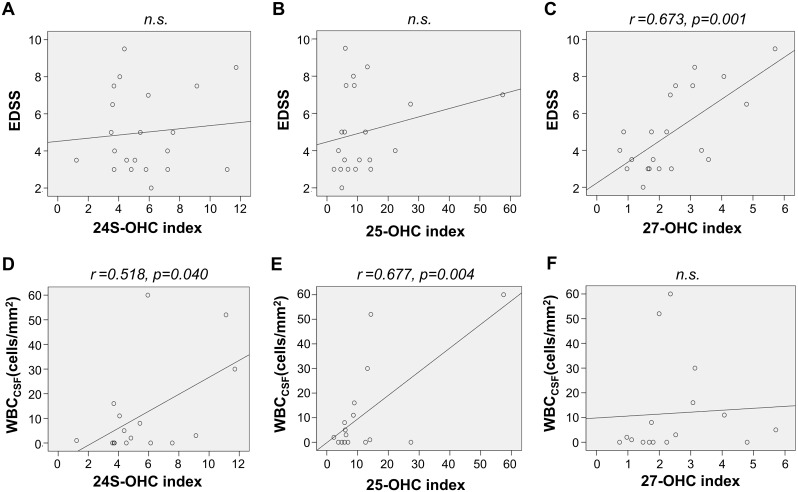
Association of the CNS-derived OHCs with disability and inflammation at acute attack of NMO. The OHC_CSF_ index was calculated to control the effects of the disruption in the BBB on the levels of these OHCs in the CSF. The associations of the OHC_CSF_ index with the disability (A–C) and number of inflammatory cells in the CNS (D–E) were assessed. The 27-OHC_CSF_ index was significantly associated with disability at acute attacks of NMO (C), moreover the 24-OHC_CSF_ index (D) and 25-OHC_CSF_ index (E) were associated with the number of the inflammatory cells in the CNS. EDSS = extended disability scale score; *n*.*s*. = not significant; OHC = hydroxycholesterol,; WBC_CSF_ = number of white blood cells in the CSF.

We also measured the OHC_CSF_ index that can control the effect of the BBB disruption on the level of OHC_CSF_, and thereby could assess the level of CNS-derived OHC. Using these OHC_CSF_ index, we assessed the association of CNS-derived OHCs with the disability of patients and also with the number of the inflammatory cells in their CSF (WBC_CSF_). The 27-OHC_CSF_ index were associated with disability (0.723; 95% CI– 0.181, 0.620; *p* = 0.002), while the 24-OHC_CSF_ index (0.518; 95% CI– 1.070, 38.121; *p* = 0.040) and 25-OHC_CSF_ index (0.677; 95% CI– 4.313, 18.532; *p* = 0.004) were associated with WBC_CSF_ in NMO patients (Fig 4).

27-OHC is synthesized mostly from cholesterol by cholesterol 27-hydroxylase (CYP27) [47]. In previous studies, 27-OHC prevented neuronal apoptosis [48] and regulated myelin-associated genes [49]. Moreover, it is the endogenous selective estrogen receptor modulator (SERM) [10] for estrogen receptor α (ERα) and β(ERβ), which can inhibit inflammation and demyelination, respectively [50,51]. Inflammation and demyelination are the two crucial molecular pathways that mediate the pathogenesis of NMO. Why 27-OHC_CSF_ is increased and associated with disease disability in patients with NMO is unclear at present. We speculate that either of following mechanisms could be responsible for it; 1) massive microgliosis [52] in NMO patients, as well as an overabundance of the CYP27 enzyme in microglia [53], could lead to the overproduction of 27-OHC_CSF_; 2) damaged astrocyte in NMO patients could cause altered de novo synthesis of cholesterol in the CNS [54], which in turn increased the net influx of the 27-OHC from the circulation [55]. The disruption of the BBB is proposed to be associated with the pathogenesis of NMO [34], which can affect the level of OHC_CSF_ in humans by increasing the diffusion of 27-OHCserum into the CNS [46]. However, the results of this study, including multivariate analysis (Table 4) and the 27-OHC_CSF_ index (Fig 4), suggest that 27-OHC_CSF_ is independently associated with the disability of patients with NMO, regardless of BBB disruption.

Though 25-OHC has been long been considered to be a strong regulator of cholesterol homeostasis [56], recent studies have shown that it is actively involved in inflammation, and can induce the expression of pro-inflammatory cytokines [11]. It can also cause mitochondria-dependent apoptosis via the generation of reactive oxygen species [57], and be a precursor of the 7α, 25-dihydroxyxcholesterol which is the most potent ligand for activation and migration of the B lymphocyte [58]. Moreover, as microglia can be a major source of 25-OHC production in the CNS [59], OHC might be the mediator of the microglia-mediated neuronal damage in demyelinating diseases of the CNS [60, 61]. Our data imply that 25-OHC could be associated with CNS inflammatory responses in NMO, by showing that the level of 25-OHC_CSF_ is increased (Fig 2) and is also associated with the number of the CNS inflammatory cells (Fig 4) in NMO patients.

24S-OHC is known to be generated mostly in the CNS [62], and conversion of cholesterol into 24S-OHC is thought to be the main route of cholesterol elimination from the brain [63, 64]. Increased levels of 24S-OHC in the CSF have been reported in neurodegenerative diseases, such as Alzheimer’s disease, and in active inflammatory diseases, such as active MS [46]. In our study, though the level of 24-OHC_CSF_ was only marginally increased, it was significantly associated with the number of CNS inflammatory cells (WBC_CSF_) in NMO patients (Fig 4). This result, together with the previous studies, could imply that 24-OHC_CSF_ might be increased in NMO, as a results of CNS damage due to inflammatory responses.

The level of OHC can be altered by a number of degenerative or inflammatory disease in the CNS [37, 62, 64]. Therefore, we could considered the possibility that the relatively high levels of OHC_CSF_ in NMO patients compared to our controls might have stem from decreased levels of OHC_CSF_ in the controls rather than that in NMO patients. However, the majority (20 out of 23) of our control patients did not have any CNS disease. Moreover, in our sub-group analysis comparing NMO patients (n = 26) with controls without CNS involvement (n = 20), the NMO patients still showed significantly higher levels of 25-OHC_CSF_ and 27-OHC_CSF_ than these controls without CNS involvements (data not shown). Therefore, we could conclude that the difference in the level of OHC_CSF_ in our NMO and controls was due to the increased level of OHC_CSF_ in NMO, rather than the decreased levels of OHC_CSF_ in controls.

There are several limitations to this study. First, the number of samples was relatively small. Second, while OHC_CSF_ levels were increased in NMO patients, and were associated with disability during acute attack and/or number of the inflammatory cells in their CSF, no causal relationship was demonstrated since this study involved human subjects. Further studies with experimental NMO models are needed to determine the causal relationship between OHC concentration and NMO. Third, though it seems to be reasonable to consider that the increased 27-OHC_CSF_ in our NMO patients was independent of BBB disruption, we cannot completely rule out the possibility that this disruption of BBB could have facilitate a minor degree of penetration of OHC_serum_ into the CNS. Fourth, we did not assess the level of other inflammatory parameters, such as cytokines or CSF glial fibrillary acidic protein [65] nor assessed their association with the level of OHCs, which could be another interesting point. Moreover, investigation into the levels of other types of OHCs, other than these 3 major OHCs, could also be another important point. Lastly, all our samples in the NMO and control groups were stored at −80°C. It seems less likely that this storage condition could interfere with our finding that OHC_CSF_ were increased in NMO patients compared to controls and associated with disability or CNS inflammation, for the following reasons; 1) all samples (NMO and controls) underwent same storage condition, 2) our storage condition was in accordant with the international biobank consent protocol [36], and 3) it had been used in a previous study on OHCs [37].

## Conclusions

In this study, two highly sensitive and selective analytical methods for the simultaneous quantitation of 24S-, 25-, and 27-OHC levels in human serum and CSF were developed. This is the first reported study to simultaneously quantify 24S-, 25-, and 27-OHC levels in serum and CSF in the human subject by LC-MS/MS. The levels of 25- and 27-OHC_CSF_ were increased during their acute attack of NMO patients. Moreover, 27-OHC_CSF_ was associated with the degree of disability, while 24-OHC_CSF_ and 25-OHC_CSF_ were associated with the number of inflammatory cells in these patients. These results imply that OHCs in the CNS might play a role in the pathogenesis of NMO, and may therefore be a potential treatment target.
